# Prolonged bowel disuse under parenteral nutrition elevates risk of acalculous cholecystitis in patients with low-output enterocutaneous fistulas

**DOI:** 10.3389/fnut.2026.1809537

**Published:** 2026-07-02

**Authors:** Tingting Shao, Risheng Zhao, Zheng Yao, Xin Xu

**Affiliations:** 1 Department of Anesthesiology, Jiangning Hospital, Nanjing, Jiangsu, China; 2 Department of General Surgery, Jiangning Hospital, Nanjing, Jiangsu, China

**Keywords:** acalculous cholecystitis, enterocutaneous fistula, intestinal disuse, parenteral nutrition, risk factor

## Abstract

**Background:**

Prolonged bowel rest during parenteral nutrition (PN) for low-output enterocutaneous fistulas (ECF) may induce acalculous cholecystitis (AAC). We evaluated the relationship between bowel disuse duration and AAC risk.

**Methods:**

This retrospective cohort study included 92 patients with low-output ECF managed with a standardized PN strategy. AAC diagnosis was based on CT criteria. Multivariable logistic regression and receiver operating characteristic (ROC) curve analyses were used to assess risk factors and determine predictive thresholds.

**Results:**

The incidence of AAC was 45.7% (42/92). In adjusted analysis, each day of bowel disuse increased AAC risk by 18% (aOR = 1.18, 95% CI: 1.05–1.32, *p* = 0.012). ROC analysis identified 48 days as the optimal cutoff (AUC = 0.68, sensitivity 66.7%, specificity 74%). Predictive accuracy improved substantially when combined with admission GGT (AUC = 0.924). Spontaneous fistula closure occurred in 65.2% of patients (60/92), with lower post-somatostatin output predicting higher closure likelihood (aHR = 0.99 per mL/day, *p* = 0.005). Longitudinal analysis showed nutritional parameters improved significantly (all *p* < 0.05) while liver enzymes concurrently elevated (all *p* < 0.05).

**Conclusion:**

Prolonged bowel disuse during PN for ECF was associated with an increased incidence of AAC, with 48 days identified as a potential monitoring threshold. The addition of admission GGT enhanced predictive performance, highlighting the need for individualized risk assessment when balancing fistula closure goals against hepatobiliary complications.

## 1 Introduction

The management of enterocutaneous fistulas (ECF) poses a significant clinical challenge, requiring a multidisciplinary approach to achieve successful closure and prevent life-threatening complications (1–3). Prior to definitive surgical intervention, optimal care typically integrates surgical expertise for source control and drainage, nutritional support to correct deficits and promote anabolism, and specialized nursing care for wound management (4). Although enteral nutrition has been widely accepted and utilized in treatment, a structured parenteral nutrition (PN) strategy combined with somatostatin analogues aims to reduce intestinal secretion and fistula output, thereby decreasing local pressure and creating conditions conducive to spontaneous closure in patients with a high likelihood of closure (5–9). This approach may not only shorten overall treatment duration but also, more importantly, avoid the substantial risks associated with complex fistula resection surgery in medically compromised patients.

However, the implementation of an exclusive PN strategy necessitates bowel rest, which introduces its own set of metabolic and physiological hazards. A critical concern is the development of acute acalculous cholecystitis (AAC)—a condition frequently observed in critically ill, metabolically stressed patients undergoing prolonged fasting (10, 11). AAC is associated with high morbidity and mortality, largely due to delayed diagnosis resulting from its often inconspicuous clinical presentation (11). Our previous research has identified prolonged bowel disuse as a primary risk factor for AAC (12). Notably, within the specific context of ECF management, the concurrent use of somatostatin—known to inhibit gallbladder contractility and promote biliary stasis—may further elevate this risk during PN therapy.

Despite the established efficacy of PN in fistula management, the specific nutritional–metabolic complication of AAC in this vulnerable population remains underexplored. Therefore, this study focuses on the impact of a PN strategy for low-output ECF on the incidence of AAC. We specifically investigated the association between the duration of bowel disuse during PN therapy and the development of AAC. The aim is to elucidate this relationship to inform risk stratification, enhance monitoring protocols, and optimize the integrated nutritional and medical management of patients with ECF.

## 2 Materials and methods

### 2.1 Patients and follow-up

This single-center retrospective cohort study was conducted in accordance with the Declaration of Helsinki. Consecutive patients referred to our tertiary care center for management of enterocutaneous fistulas (ECF) and treated with a defined PN strategy between January 2022 and December 2025 were screened for eligibility. Exclusion criteria were: (1) prior cholecystectomy or gallbladder puncture; (2) concomitant cholelithiasis at admission; (3) inflammatory bowel disease affecting fistula closure; and (4) evidence of active malignancy. The primary outcome was the occurrence of AAC. Secondary outcomes included: (1) rate of spontaneous fistula closure; and (2) longitudinal trends in body mass index (BMI), albumin, prealbumin, and liver enzymes [total bilirubin, alanine aminotransferase (ALT), aspartate aminotransferase (AST), glutamyl transferase (GGT)].

### 2.2 Parenteral nutrition strategies

The PN strategy was indicated for patients with low-output ECF ( < 500 mL/day) (9), absent abdominal infection or peri-fistulous abscess on imaging, and without complete gastrointestinal discontinuity. The protocol comprised three components:

(1) Bowel rest and secretion control

- lNasogastric decompression
- lStrict fasting with water restriction
- lContinuous somatostatin infusion (12 mg/day)

(2) Nutritional anf metabolic support

- lTotal PN providing 30 kcal/kg/day
- lConcomitant intravenous hydration to maintain urine output ≥ 1,000 mL/day

(3) Monitoring

- lLaboratory assessment (complete blood count and biochemistry) every 3–4 days
- lFistula output monitored daily

### 2.3 Transition to enteral nutrition

Upon cessation of drainage for seven consecutive days, a contrast study was performed to confirm spontaneous closure. If confirmed, enteral nutrition (Peptisorb^®^ via nasogastric tube, 30 kcal/kg/day) was gradually reintroduced, given that the prolonged disuse of the digestive tract favored a formula with reduced gastrointestinal irritation and enhanced absorption (13). If closure was not achieved after 30 days, enteral nutrition was still initiated to optimize nutritional status for potential future surgery, while acknowledging the possibility of delayed spontaneous closure.

### 2.4 Diagnose of AAC

AAC was suspected based on clinical criteria (new-onset SIRS, unexplained abdominal pain, vomiting, or positive Murphy’s sign). According to the Tokyo Guidelines 2018 (TG18), imaging findings are a required diagnostic component for acute cholecystitis. Diagnosis was confirmed by CT showing gallbladder distension with wall thickness > 3 mm (12). While ultrasound is an alternative, CT was preferred in our ECF population due to abdominal wounds/ostomies and the need to exclude other intra-abdominal pathologies.

Once AAC was diagnosed, all patients initially received conservative medical therapy (14), including: (1) broad-spectrum antibiotics (a third-generation cephalosporin, e.g., ceftriaxone or cefoperazone); (2) supportive care (intravenous fluid resuscitation, electrolyte repletion, and analgesia as needed); and (3) strict fasting to reduce the pressure in the biliary system. Percutaneous cholecystostomy was performed if any of the following criteria were met after 48 h of conservative treatment: (a) persistent or worsening systemic inflammatory response syndrome (SIRS); (b) new-onset septic shock or multiorgan dysfunction; or (c) worsening abdominal pain or localized peritonism.

### 2.5 Data collection and statistical analysis

Continuous variables are presented as median (interquartile range, IQR) and categorical variables as frequency (%). For the primary outcome, univariate followed by multivariable logistic regression identified independent risk factors for AAC. The functional form of the association between bowel disuse duration and AAC was examined using linear and restricted cubic spline (RCS, 3 knots) models, with model selection informed by the Akaike Information Criterion (AIC). Diagnostic performance was evaluated via receiver operating characteristic (ROC) curve analysis; the optimal cutoff was determined by maximizing Youden’s index. Spontaneous closure was analyzed using Cox proportional hazards regression. Longitudinal changes in nutritional and hepatic parameters were assessed with linear mixed-effects models regression, modeling the parameter value against time. A two-sided *P*-value < 0.05 defined statistical significance. Analyses were performed using R software (v4.3.0).

## 3 Results

### 3.1 Patient characteristics

After applying exclusion criteria (4 prior cholecystectomy or gallbladder puncture, 3 pre-existing gallstones, 8 inflammatory bowel disease, and 2 active malignancies), 92 patients were included in the final analysis. AAC developed in 42 patients (45.7%). Notably, all cases manifested during the early phase of enteral nutrition reintroduction following PN; no case occurred during ICU stay. The characteristics of the 92 enrolled patients represented in Table 1.

**TABLE 1:** Characteristics of the enrolled 92 patients at admission

Characteristics	Total cohort
Demographic data	
Male, no. (%)	52 (56.5)
Age, years (median, IQR)	46 (34–58)
BMI, kg/m^2^ (median, IQR)	21.3 (20.3–22.3)
Fistula characteristics at admission	
Output volume, mL (median, IQR)	300 (200–400)
Output volume after somatostatin initiation, mL (median, IQR)	110 (70–160)
Number of drainage tubes (median, IQR)	1 (1–1)
NRS 2002 ≥ 3; no. (%)	51 (55.4)
Interval from fistula diagnosis to PN initiation, days (median, IQR)	29 (25–32)
Etiology, no. (%)	
Adhesive intestinal obstruction	35 (38.0)
Radical tumor surgery	34 (37)
Trauma	20 (21.7)
Thrombotic intestinal obstruction	3 (3.3)
Location, no. (%)	
Upper gastrointestinal tract	22 (23.9)
Small intestine	38 (41.3)
Ileocolonic anastomosis	18 (19.6)
Colon	14 (15.2)
SOFA at admission (median, IQR)	3 (2–4)
Mean arterial pressure < 65 mmHg, no. (%)	32(34.8)
ICU stay after admission, days (median, IQR)	6 (4–8)
Antibiotics**, no. (%)	66 (71.7)
Opioid, no. (%)	0
Hemoglobin, g/L (median, IQR)	112 (99–121)
Albumin, g/L (median, IQR)	33 (31–36)
Prealbumin, g/L (median, IQR)	23 (20–27)
White blood cell, 10^∧^9 (median, IQR)	7.1 (5.4–8.6)
CRP, mg/L (median, IQR)	24 (17–32)
Bilirubin, mmol/L (median, IQR)	17.3 (12–20.7)
Creatinine, mmol/L (median, IQR)	52 (42–61)
ALT, U/L (median, IQR)	53 (36–76)
AST, U/L (median, IQR)	67 (52–81)
GGT, U/L (median, IQR)	110 (68–147)
Bowel disuse duration, days (median, IQR)*	47 (40–52)
Chronic hepatitis, no. (%)	2 (2.2)

*For urinary tract infections and lung infections, rather than abdominal infections.

**We calculate Duration of bowel disuse as Interval from diagnosed fistula to PN initiation+duration of parenteral nutrition strategy.

The cohort was predominantly male (56.5%), with a median age of 46 years (IQR 34–58) and BMI of 21.3 kg/m^2^ (IQR 20.3–22.3). The median interval from fistula diagnosis to admission was 29 days (IQR 25–32). Median fistula output was 300 mL/day (IQR 200–400). Primary etiologies were adhesive obstruction (38.0%), sequelae of radical tumor surgery (37.0%), trauma (21.7%), and thrombotic obstruction (3.3%).

### 3.2 Association with bowel disuse

**FIGURE 1:**
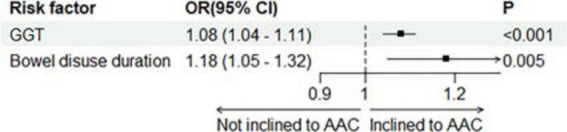
risk factors associated with AAC.

**FIGURE 2:**
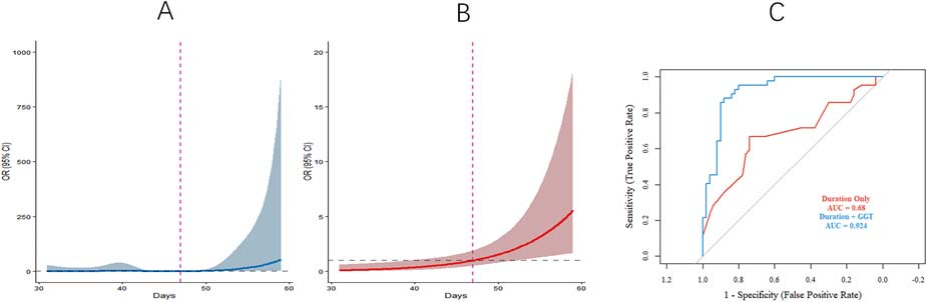
Analysis of the relationship between bowel disuse duration and AAC risk. **(A)** The non-linearity test was non-significant (*P* = 0.304) in the restricted cubic spline models using 47 days as the reference point. **(B)** The linear relationship between bowel disuse duration and the predicted odds of AAC. **(C)** Receiver operating characteristic (ROC) curve analysis showing the diagnostic performance of bowel disuse duration for predicting AAC (AUC = 0.68, 95% CI: 0.57–0.79). The optimal cut-off value was determined to be 48 days.

No death was detected in the AAC patients. Characteristics and outcomes of AAC are detailed in Table 2. Univariate analysis (Table 3) identified admission GGT level and bowel disuse duration as significant risk factors. In a multivariable model adjusted for admission GGT, bowel disuse duration remained an independent predictor [adjusted odds ratio (aOR) per day = 1.18, 95% CI: 1.05–1.32, *P* = 0.012] (Figure 1). Both linear and RCS models were fitted to this association. The non-linearity test for the RCS model was non-significant (*P* = 0.304), and the linear model demonstrated a slightly better fit according to the Akaike Information Criterion (AIC: 69.21 vs. 71.16 for the linear and RCS models, respectively; Figures 2a,b). As illustrated in Figure 2B, the predicted odds ratio for AAC increased linearly with each additional day of bowel disuse, using 47 days as the reference point.

**TABLE 2:** Characteristics of AAC in the 42 patients.

Characteristics	Number of patients
Signs	
Murphy’s sign, no. (%)	8 (19.1)
Upper abdominal tenderness, no. (%)	22 (52.4)
Rebound tenderness, no. (%)	5 (11.9)
Jaundice, no. (%)	9 (21.4)
Symptoms	
Vomit, no. (%)	24 (57.1)
Fever, no. (%)	33 (78.6)
Upper abdominal pain, no. (%)	18 (42.9)
Timing of occurred AAC	
Resume EN, no. (%)	42 (100)
Days after EN initiation, days (median, IQR)	8 (4–11)
ALT when diagnosis of cholecystitis, U/L (median, IQR)	89 (69–111)
AST when diagnosis of cholecystitis, U/L (median, IQR)	96 (68–109)
GGT when diagnosis of cholecystitis, U/L (median, IQR)	149 (108–168)
Bilirubin when diagnosis of cholecystitis, mmol/L (median, IQR)	19 (14–24)
Treatment	
Conservative treatment, no. (%)	6 (14.3)
Percutaneous cholecystostomy, no. (%)	36 (85.7)
Bile culture results (can be displayed only in patients received Percutaneous cholecystostomy, *n* = 36)	
Klebsiella pneumoniae, no. (%)	12 (33.3)
*Escherichia coli*, no. (%)	18 (50)
Enterococcus faecium, no. (%)	6 (16.7)

**TABLE 3:** Unadjusted analysis for AAC.

Characteristics	AAC (*n* = 42)	Non-AAC (*n* = 50)	*P*
Demographic data			
Male, no. (%)	21 (50)	31 (62)	0.25
Age, years (median, IQR)	47 (38–60)	44 (33–56)	0.26
BMI, kg/m^2^ (median, IQR)	21.2 (20.1–22.3)	21.3 (20.5–22.3)	0.68
Fistula characteristics at admission			
Output volume, mL (median, IQR)	300 (200–400)	300 (100–400)	0.28
Output volume after somatostatin initiation, mL (median, IQR)	110 (78–180)	110 (70–140)	0.25
Number of drainage tubes (median, IQR)	1 (1–1)	1 (1–1)	0.72
NRS 2002 ≥ 3; no. (%)	25 (59.5)	26 (52)	0.47
Interval from fistula diagnosis to admission, days (median, IQR)	29 (26–32)	29 (25–32)	0.82
Etiology, no. (%)			0.51
Adhesive intestinal obstruction	13 (30.9)	22 (44)	
Radical tumor surgery	16 (38.1)	18 (36)	
Trauma	11 (26.2)	9 (18)	
Thrombotic intestinal obstruction	2 (4.8)	1 (2)	
Location, no. (%)			0.48
Upper gastrointestinal tract	9 (21.4)	13 (26)	
Small intestine	21 (50)	17 (34)	
Ileocolonic anastomosis	7 (16.7)	11 (22)	
Colon	5 (11.9)	9 (18)	
SOFA at admission (median, IQR)	3 (2–4)	3 (2–4)	0.68
Mean arterial pressure < 65 mmHg, no. (%)	16 (38.1)	16 (32)	0.54
ICU stay after admission, days (median, IQR)	6 (4–8)	5 (3–8)	0.021**
Antibiotics, no. (%)	32 (76.2)	34 (68)	0.38
Opioid, no. (%)	0	0	-
Hemoglobin, g/L (median, IQR)	110 (95–121)	113 (99–120)	0.36
Albumin, g/L (median, IQR)	32 (30–36)	33 (31–36)	0.26
Prealbumin, g/L (median, IQR)	23 (20–26)	24 (21–27)	0.51
White blood cell, 10∧9 (median, IQR)	7.1 (5.4–8.4)	7.3 (5.3–8.7)	0.77
CRP, mg/L (median, IQR)	26 (17–32)	22 (16–31)	0.32
Bilirubin, mmol/L (median, IQR)	18.1 (9.8–20.6)	17.3 (12.5–19.7)	0.97
Creatinine, mmol/L (median, IQR)	52 (42–58)	55 (41–62)	0.51
ALT, U/L (median, IQR)	55 (42–76)	52 (33–76)	0.25
AST, U/L (median, IQR)	67 (52–82)	67 (52–76)	0.81
GGT, U/L (median, IQR)	152 (121–186)	72(61–104)	< 0.001
Bowel disuse duration, days (median, IQR)*	50 (44–55)	42 (38–51)	0.010**
Chronic hepatitis, no. (%)	1 (2.4)	1 (2)	0.90

*We calculate Duration of bowel disuse as Interval from diagnosed fistula to PN initiation+duration of parenteral nutrition strategy.

**Considering that ICU stay is correlated with digestive tract deadaptation, we included the cases with a smaller *p*-value for digestive tract deadaptation in the multivariate analysis.

**FIGURE 3:**
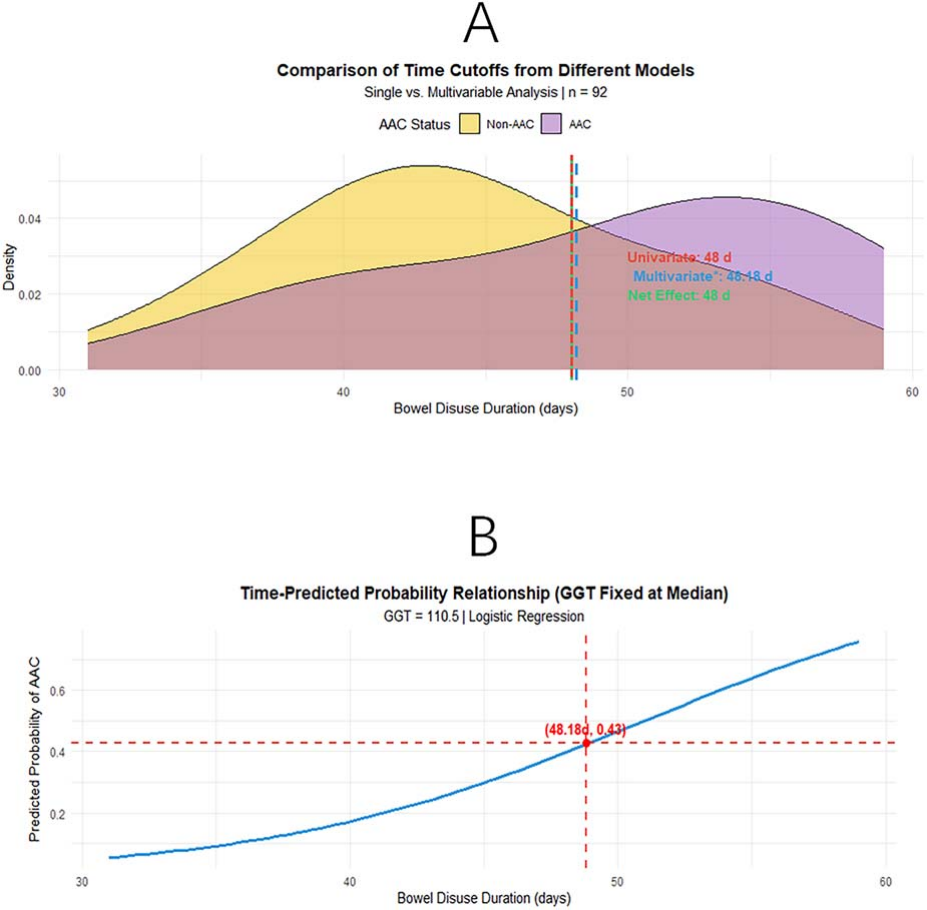
comparative analysis of bowel disuse duration cutoffs. **(A)** comparison of time cutoffs from different models. **(B)** Time-predicated probability relationship (GGT fixed at median).

The diagnostic performance of bowel disuse duration for predicting AAC was evaluated using ROC curve analysis, which yielded an AUC of 0.68 (95% CI: 0.57–0.79, Figure 2C), indicating low-to-moderate discriminatory ability (AUC lying within the range of 50–70%). The optimal cut-off value, determined by maximizing Youden’s index, was 48 days. At this threshold, the sensitivity and specificity were 66.7 and 74%, respectively, with positive and negative predictive values of 68.3 and 72.5%. The positive likelihood ratio was 2.56, while the negative likelihood ratio was 0.45. After adjusted for GGT, the optimal cutoff for bowel disuse duration in predicting AAC was approximately 48 days (48.18 days) as well (Figure 3). Its combination with admission GGT level substantially improved predictive performance (AUC = 0.924; *P* < 0.001; Figure 2C).

### 3.3 Spontaneous closure

**FIGURE 4:**
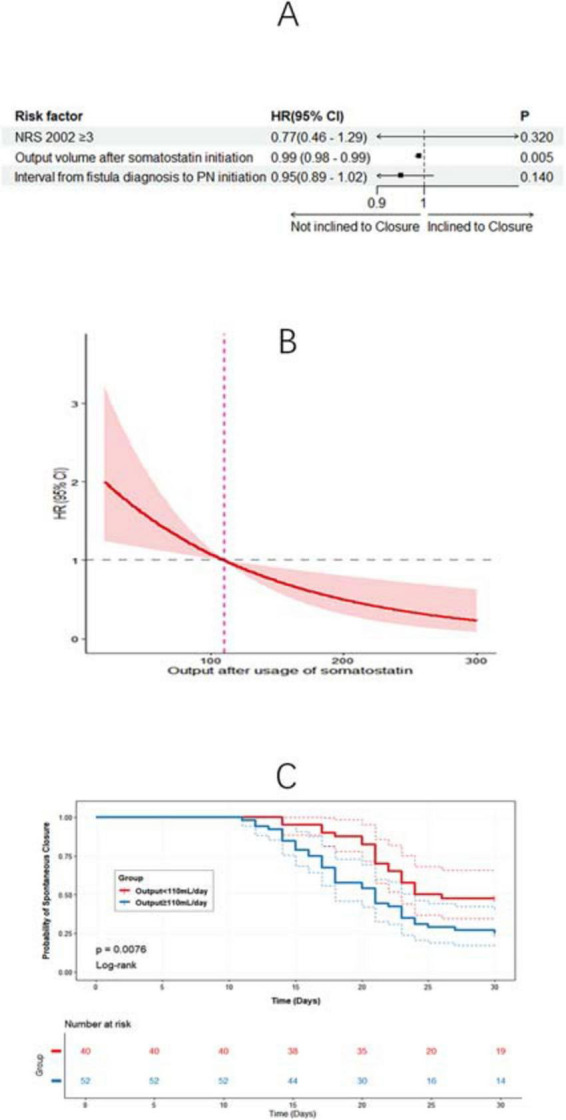
Impact of fistula output after somatostatin initiation on spontaneous closure. **(A)** Multivariable Cox regression analysis showing that lower fistula output was the sole independent predictor of closure. **(B)** The linear relationship between output after somatostatin initiation. **(C)** Kaplan-Meier curve comparing closure probability between patients with fistula output ≤110 mL/day and > 110 mL/day after somatostatin initiation (Log-rank *P* = 0.0078).

Spontaneous closure occurred in 60 patients (65.2%) at a median of 20 days (IQR 17–23). In multivariable Cox regression (adjusted for significant univariate variables), lower fistula output after somatostatin initiation was the sole independent predictor of closure [adjusted hazard ratio (aHR) per mL/day = 0.99, 95% CI: 0.98–0.99, *P* = 0.005, Figure 4A]. Its non-linear component was not significant in RCS model (*P* = 0.256, Figure 4B), suggesting an approximately linear relationship. Using the median as a reference, patients with output after somatostatin initiation ≤ 110 mL/day had a significantly higher probability of closure (Log-rank *P* = 0.0078, Figure 4C).

### 3.4 Longitudinal trends in BMI, albumin, prealbumin, and hepatic parameters

**FIGURE 5:**
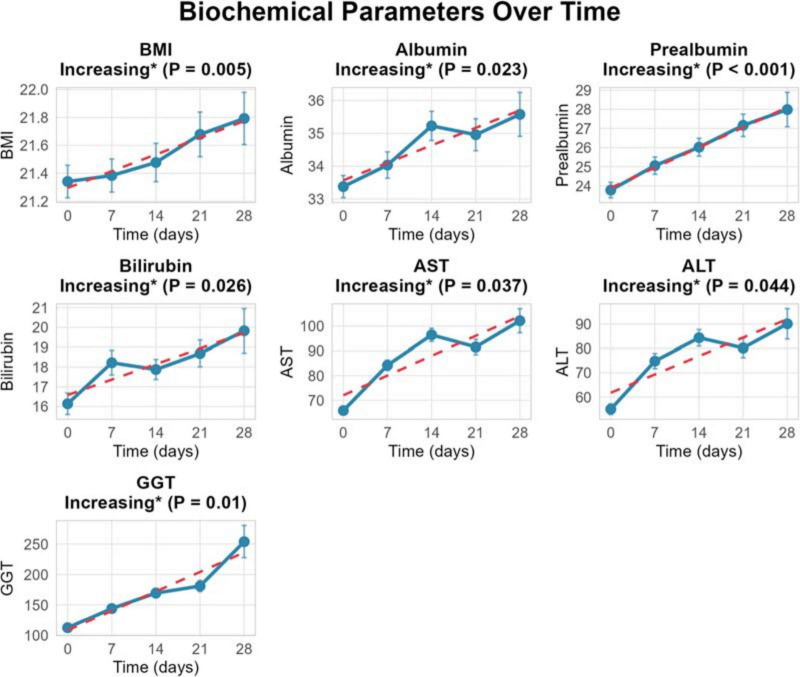
Longitudinal trends of nutritional and hepatic parameters during the observation period.

As depicted in Figure 5, linear mixed-effects models confirmed statistically significant increasing trends for BMI (*P* = 0.005), albumin (*P* = 0.023), and prealbumin (*P* < 0.001). Liver enzymes exhibited significant elevation (AST: *P* = 0.037; ALT: *P* = 0.044; GGT: *P* = 0.01), with bilirubin also showing an upward trend (*P* = 0.026). These parallel upward trends carry opposing clinical implications—improving metabolic status versus worsening hepatobiliary injury—highlighting the complex interplay between clinical recovery and hepatic response during the observation period.

## 4 Discussion

Our study reveals a high incidence (45.7%) of AAC among patients receiving structured PN for low-output ECF. Crucially, the duration of bowel disuse during PN therapy was identified as an independent, linear risk factor for AAC. We established a clinically actionable cutoff of approximately 48 days, beyond which risk escalates significantly, particularly when combined with elevated admission GGT levels. Concurrently, the PN strategy demonstrated efficacy, achieving a 65.2% spontaneous fistula closure rate, primarily predicted by low post-somatostatin output. Longitudinal data highlighted a divergent response: significant improvement in nutritional parameters alongside a marked elevation in liver enzymes, underscoring the complex hepatobiliary impact of this therapeutic approach.

The central novelty of this work lies in the quantification of a specific, modifiable risk exposure—bowel disuse duration—and the proposal of a concrete time-based cutoff (48 days) for heightened clinical vigilance regarding AAC. While AAC is a recognized complication of critical illness and prolonged fasting (14, 15), its epidemiology within the precise paradigm of intentional bowel rest for ECF management has been poorly defined. Our reported incidence of 45.7% is strikingly high, far exceeding rates reported in general ICU populations. This underscores the unique vulnerability of the ECF patient undergoing PN. AAC carries substantial morbidity and mortality (12, 14–16), often attributed to diagnostic delays owing to its insidious, non-specific presentation (e.g., unexplained fever, ileus, or sepsis) in already complex, postoperative patients (15, 16), as reflected in our cohort’s symptom profile (12, 14). The pathophysiology of AAC is multifactorial, centered on gallbladder stasis and ischemia. Stasis, leading to bile concentration and sludge formation, is driven by lack of enteral stimulation (and thus reduced cholecystokinin release) and by pharmacological agents that inhibit gallbladder contractility, such as somatostatin analogs (17). Ischemic injury to the gallbladder wall can result from low-flow states, systemic inflammation, and increased biliary pressure (18).

Our findings seamlessly align with this pathophysiological framework and elucidate why the PN strategy for ECF creates a “perfect storm” for AAC. The risk operates through two synergistic mechanisms inherent to the patient population and the therapy itself. First, the therapeutic cornerstone is deliberate, prolonged bowel disuse. This directly induces gallbladder atony and profound biliary stasis, a risk we have now quantified. Second, the underlying patient substrate is inherently compromised. Patients developing ECF, typically after major abdominal surgery for conditions like adhesive obstruction or malignancy, are frequently in a state of pronounced metabolic stress, characterized by anemia, hypoalbuminemia, and systemic inflammation (19). This baseline vulnerability impairs tissue perfusion and repair mechanisms, predisposing the gallbladder mucosa to ischemic injury even in the absence of overt shock. The concurrent administration of somatostatin, while effective for reducing fistula output, potently exacerbates biliary stasis by inhibiting gallbladder emptying (20). Thus, the PN strategy, while addressing one critical problem (the fistula), inadvertently amplifies the risk for another (AAC) by intensifying both key pathological drivers: stasis and susceptibility to ischemia.

It is imperative to adopt a balanced perspective on the PN strategy. Our study confirms its significant benefits. The 65.2% spontaneous closure rate is commendable and can spare a fragile patient cohort the high risks of definitive surgical repair. Furthermore, the consistent and significant improvements in BMI, albumin, and prealbumin demonstrate the strategy’s effectiveness are fundamental to healing and overall recovery. However, the observed significant elevations in AST, ALT, GGT, and bilirubin serve as an important counterpoint. This hepatic biochemical response, likely reflecting cholestasis, hepatic steatosis, or drug-induced effects from PN and somatostatin, is not merely a laboratory aberration. It provides a physiological echo to the clinical emergence of AAC, highlighting a spectrum of hepatobiliary dysfunction triggered by the therapeutic regimen. Therefore, the clinician must view this strategy through a dual lens: a powerful tool for fistula management and a significant perturbation of hepatobiliary physiology.

Including GGT raised AUC from 0.68 to 0.924. While bowel disuse duration captures gallbladder stasis due to lack of enteral stimulation, GGT serves as a biomarker of baseline cholestasis or hepatobiliary dysfunction. Patients with elevated admission GGT are likely more susceptible to biliary sludge formation and gallbladder inflammation when subjected to prolonged fasting and somatostatin therapy. Thus, combining a dynamic exposure (bowel disuse duration) with a baseline susceptibility marker (GGT) provides a more complete risk assessment than either factor alone. At median GGT (110 U/L), the bowel disuse cutoff was 48.18 days, confirming the 48-day threshold. Patients with GGT > 110 U/L and bowel disuse > 48 days are very high-risk.

Based on these findings, we propose a preliminary risk stratification framework for AAC in patients receiving PN for low-output ECF, acknowledging that external validation is required before widespread clinical adoption. Bowel disuse duration > 48 days alone warrants increased clinical vigilance, with a low threshold for abdominal imaging (e.g., ultrasound or CT) in the presence of any new symptoms. The combination of bowel disuse > 48 days and admission GGT > 110 U/L defines a very high-risk subgroup that should trigger intensified surveillance (e.g., weekly gallbladder ultrasound) or early prophylactic cholecystostomy. For patients with GGT ≤ 110 U/L, the 48-day cutoff remains relevant but with lower absolute risk, though clinical vigilance should still be maintained. To mitigate AAC risk, especially in patients high risk, we suggest early introduction of minimal enteral nutrition (10–20 mL/h of semi-elemental formula), periodic cycling of PN (e.g., 12 h off, 12 h on), and vigilant management of dehydration and anemia.

The use of somatostatin warrants particular attention. While it promotes fistula closure by reducing intestinal secretions, it simultaneously exacerbates biliary stasis through potent inhibition of gallbladder contractility—creating a genuine therapeutic dilemma. Given its contribution to a 65.2% spontaneous closure rate in our cohort, we suggest continuing somatostatin for the first 2–4 weeks to maximize closure probability. Beyond this period, if closure has not occurred and the patient approaches the 48-day threshold—especially with GGT > 110 U/L—risk-benefit reassessment is warranted. Options include stepwise dose reduction (e.g., from 12 to 6 mg/day), intermittent cessation (e.g., 2 days off per week), or substitution with octreotide at a lower dose, with close monitoring of fistula output for any significant increase ( > 20% rise over 48 h).

Several limitations of this study must be acknowledged. Its retrospective, single-center design carries inherent risks of selection bias and unmeasured confounding. The sample size, while substantial for a focused ECF study given the relative rarity of this condition, may limit generalizability and the precision of subgroup analyses. The diagnosis of AAC relied primarily on CT criteria; although consistent with TG18 and clinically practical in this population with abdominal wounds and ostomies, this approach may not capture very early or subclinical cases. The 48-day threshold is exploratory and requires external validation before clinical implementation. The observation that all AAC cases manifested during the early phase of enteral nutrition reintroduction is intriguing but warrants prospective validation to clarify whether refeeding itself constitutes a specific physiological stressor. Importantly, our protocol of deliberate bowel rest differs from current guidelines favoring early enteral nutrition when feasible. However, ECF managed with an intentional bowel rest strategy to maximize spontaneous closure represents a specific clinical scenario; our findings therefore apply only to this selected population and should not be generalized to patients who can safely tolerate early enteral feeding. Importantly, the high cholecystostomy rate (36/42, 85.7%) does not indicate an interventional bias. It reflects the natural consequence of our PN strategy—prolonged bowel rest—which inherently promotes gallbladder stasis and predisposes to AAC. Once AAC developed under this protocol, conservative therapy failed in the vast majority of cases, and drainage was performed strictly according to TG18 criteria (persistent or worsening clinical deterioration, not imaging alone). Thus, our approach is guideline-concordant care, not overtreatment, in a high-risk population with limited physiological reserve. External validation in less severely ill ECF populations is needed. Despite these limitations, this study provides clinically relevant evidence linking the duration of bowel disuse to a serious, potentially modifiable complication in a high-risk ECF population.

## 5 Conclusion

A structured PN strategy for low-output ECF effectively promotes fistula closure and nutritional recovery but carries a nearly 50% risk of AAC. Prolonged bowel disuse (>48 days) and elevated admission GGT were identified as independent predictors of AAC in this retrospective analysis, though causality cannot be established given the observational design.

## Data Availability

The raw data supporting the conclusions of this article will be made available by the authors, without undue reservation.
